# Supplementary material for: Microglia and amyloid precursor protein coordinate control of transient Candida cerebritis with memory deficits
Source: Nat Commun. 2019 Jan 4;10:58. doi: 10.1038/s41467-018-07991-4 (PMC6320369; doi:10.1038/s41467-018-07991-4)
Supplement: Supplementary file 1 — Supplementary Information [file 41467_2018_7991_MOESM1_ESM.pdf]

Microglia and Amyloid Precursor Protein Coordinate Control of Transient *Candida* Cerebritis With Memory Deficits

Wu, et al.

Supplementary Figures

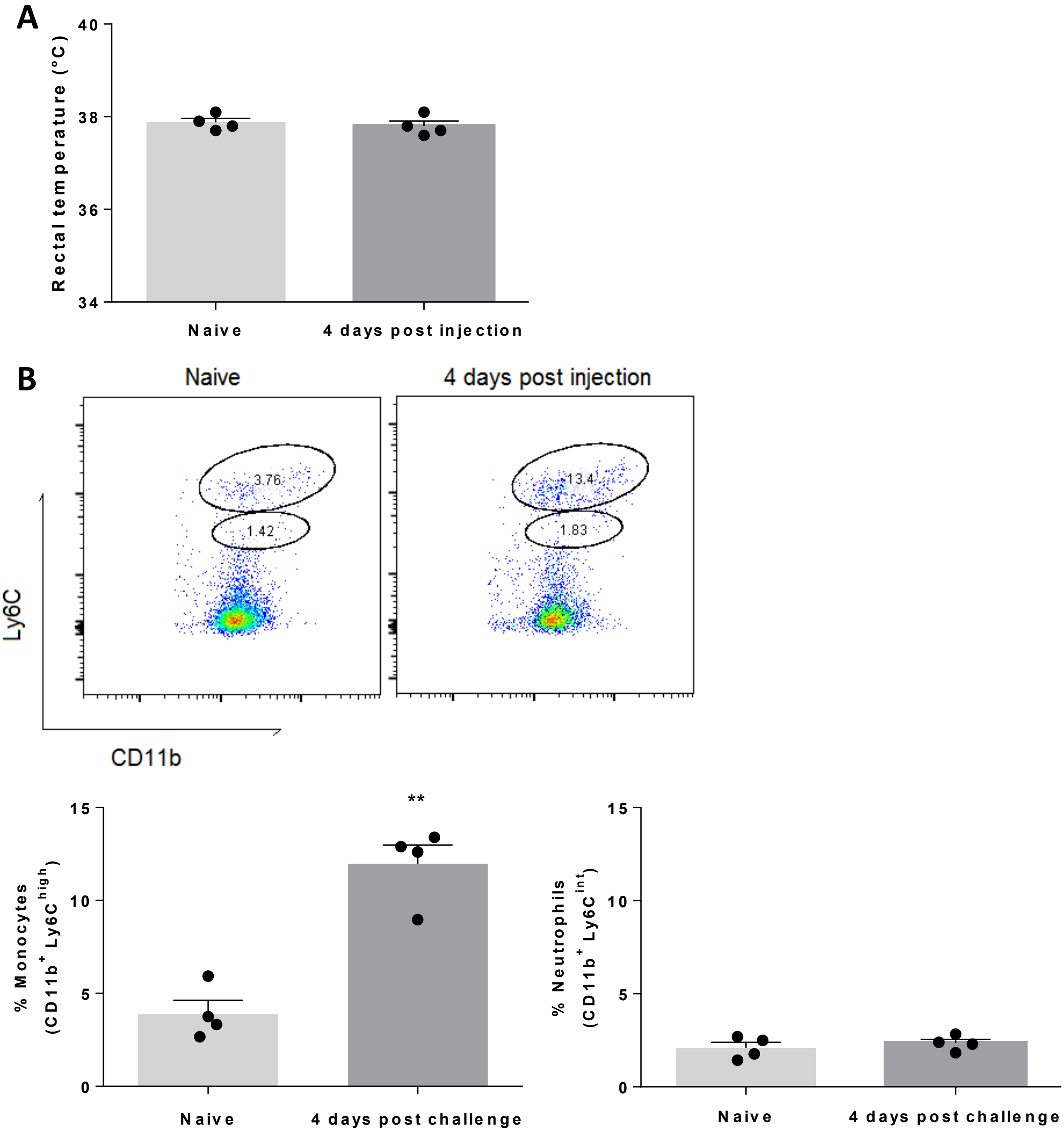

Supplementary Figure 1: Effect of low-grade fungemia on body temperature and CNS inflammation.

Four days after intravenous injection of 25,000 *C. albicans* cells, **(A)** rectal temperature was recorded. **(B)** Representative flow plots of total lymphocytes extracted from brain of naïve or challenged mice. Percentage of $CD11b^{+}$ $Ly6C^{high}$

monocytes and $CD11b^+$ $Ly6C^{int}$ neutrophils are demonstrated below. (n=4, mean ± S.E.M, **p<0.01, using two-tailed Student's t-test.)

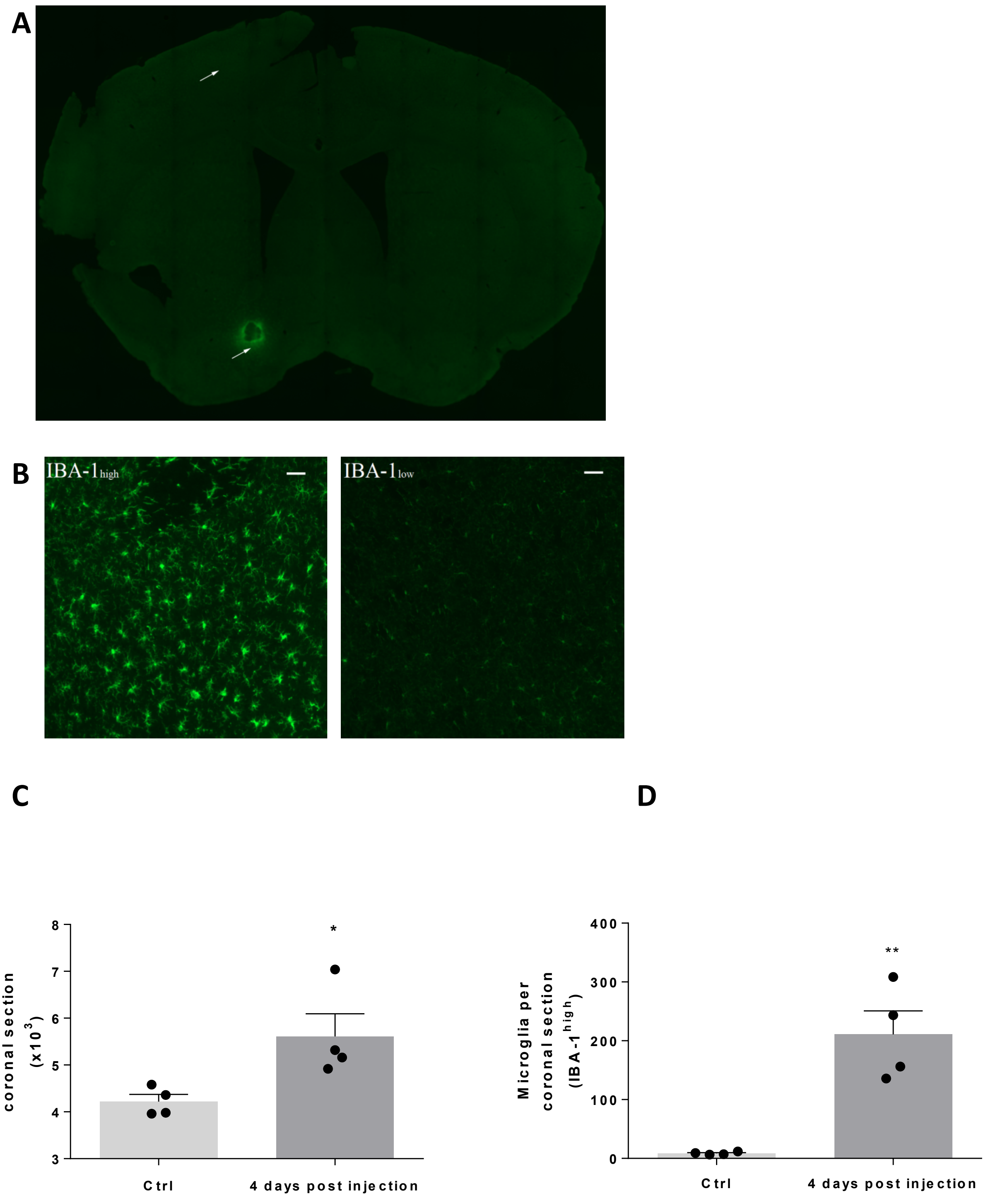

Supplementary Figure 2: Microglia count in coronal sections of brains from challenged mice.

Brains were isolated and sectioned from mice four days after intravenous injection of 25,000 *C. albicans* cells. (**A)** Low power image from a coronal brain section stained for IBA-1 revealing a single FIGG (rounded structure near bottom). **(B)** Pictures of designated areas in A which represents IBA-1_{low} resting microglia (arrow near top, panel **A**) and IBA-1_{high} activated microglia (arrow near bottom, panel **A**). (C, D) Quantification of total (C) or activated (D) microglia per coronal section. (n=4 mice per group, 9 coronal sections per mouse, mean ± S.E.M, *p<0.05, **p<0.01, using two-tailed Student's t-test.)

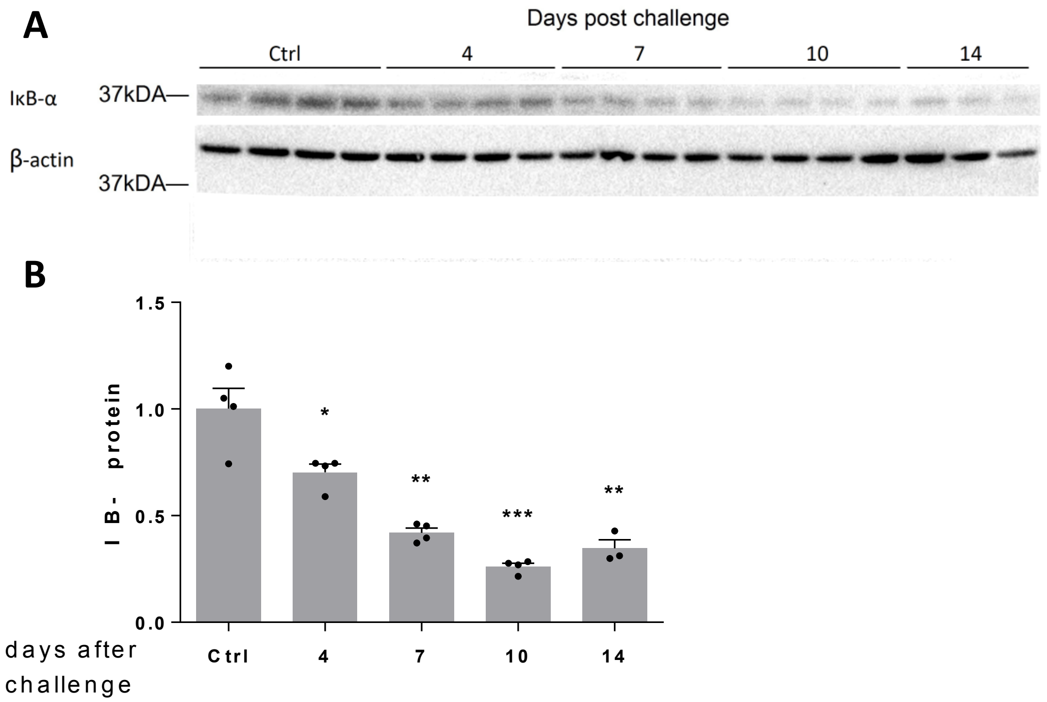

Supplementary Figure 3: IκB-α protein level in brains of mice post challenge.

Brains were isolated from mice challenged as stated before and total protein was extracted. (A) Western blot analysis of IκB-α over 14 days. (B) Densitometric quantification of the western blot data (n=3 or 4, mean ± S.E.M, *p<0.05, **p<0.01, ***p<0.001, using one-way ANOVA followed by Dunnett's test for multiple comparison.)

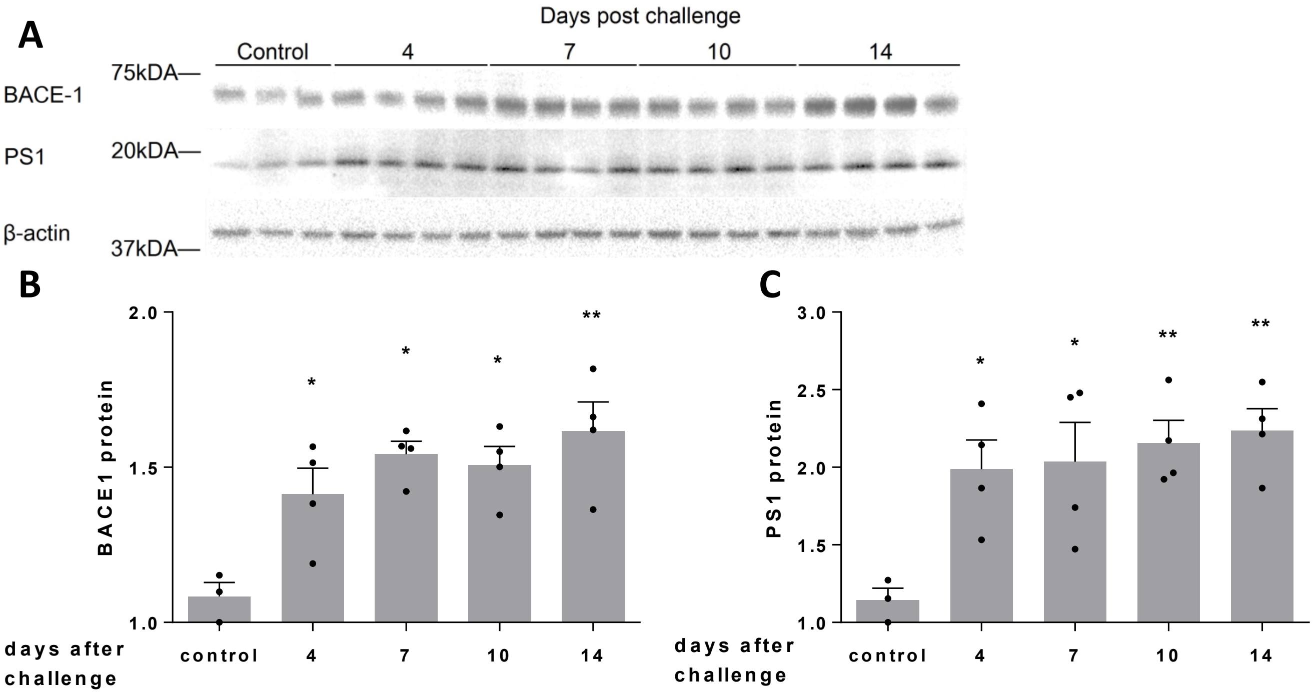

Supplementary Figure 4: BACE-1 and PSEN-1 protein level in brains of mice post challenge.

Brains were isolated from mice challenged as stated before and total protein was extracted. **(A)** Western blot analysis of BACE-1 and PSEN-1 (PS1) over 14 days. **(B, C)** Densitometric quantitation of the western blot data (n=3 or 4, mean ± S.E.M, *p<0.05, **p<0.01, using one-way ANOVA followed by Dunnett's test for multiple comparison.)

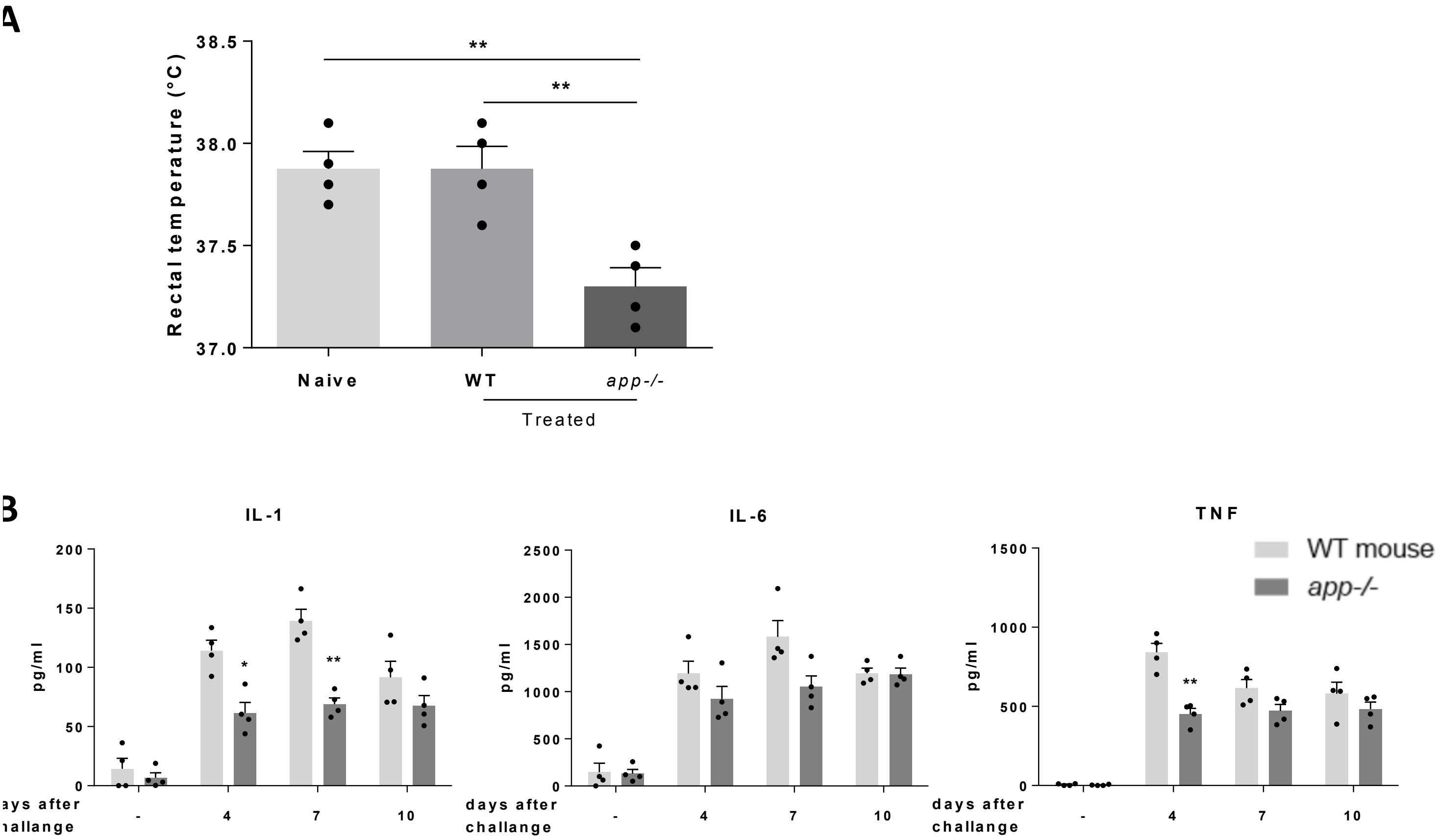

Supplementary Figure 5: Inflammation induced by *C. albicans* in *app-/-* mice.

Wildtype (WT) and *app*-/- mice were kept naïve or treated i.v. with 25,000 CFU of *C. albicans* after which whole brains were harvested at the indicated days. (A) Rectal temperature of mice four days after intravenous injection. (B) IL-1β, IL-6, and TNF cytokine levels from brain homogenates as assessed by ELISA. (n⩾4, mean ± S.E.M, *p<0.05, **p<0.01, ***p<0.001, using two-tailed Student's t-test or one-way ANOVA followed by Tukey's test for multiple comparison.)

A

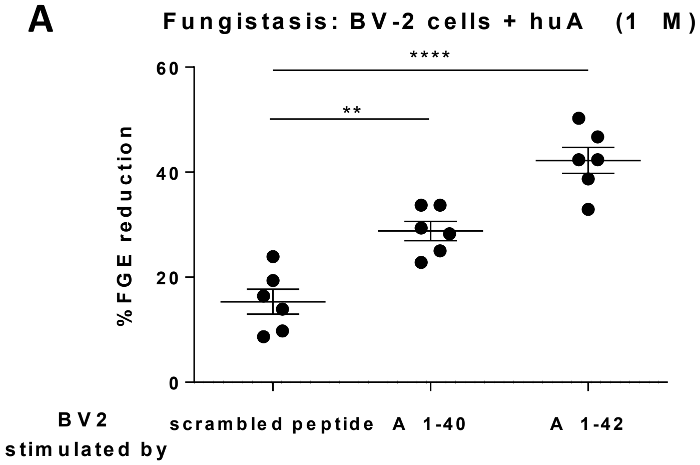

Supplementary Figure 6: Effect of human Aβ on fungistatic activity of BV2 cells.

(**A)** BV-2 cells were pre-treated with the above peptides at 2μg/ml for 6 hours and then *C. albicans* (200 viable cells/ml) were added. Fungal inhibition was calculated as in Figure 5. (n=6, mean ± S.E.M, **p<0.01, ****p<0.0001, using one-way ANOVA followed by Dunnett’s test for multiple comparison.)

A **Figure 2B**

P65

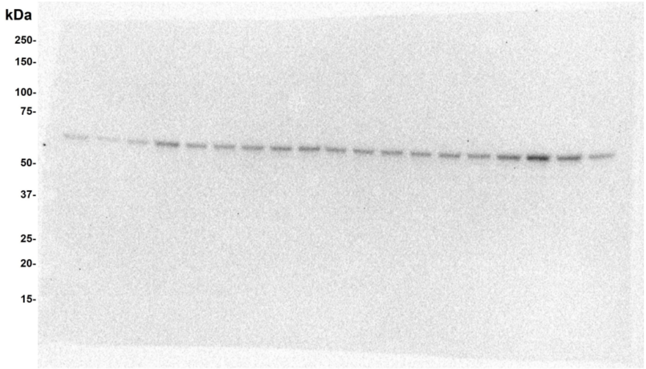

β-actin

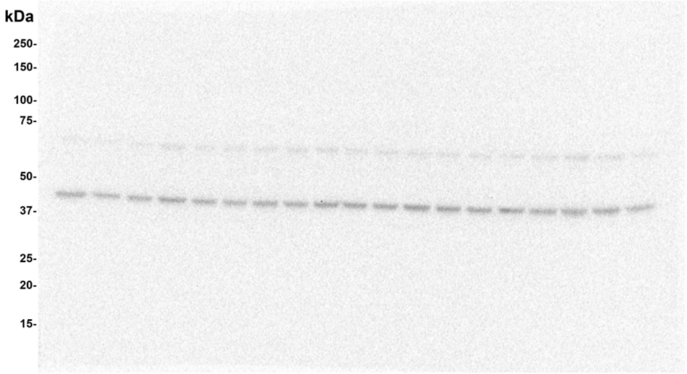

B **Figure 3B**

APP/β-actin

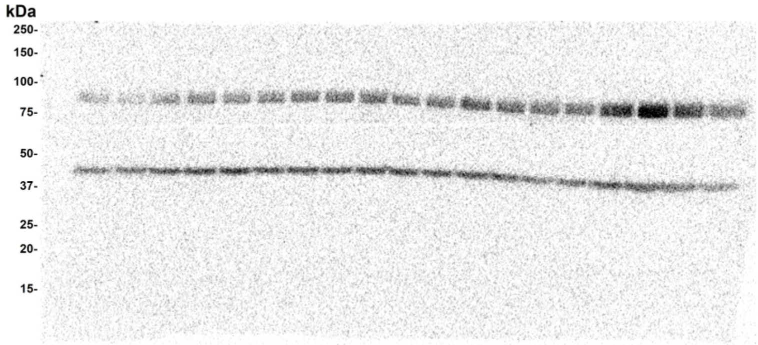

Supplementary Figure 7: Uncropped western blot images

(A) Uncropped western blot images for Figure 2B: P65 and β-actin. (B) Uncropped western blot image for Figure 3B: Amyloid precursor protein (APP) and β-actin.
